# Comparison of Five Protocols of Estrous Synchronization on Reproductive Performance of Hu Sheep

**DOI:** 10.3389/fvets.2022.843514

**Published:** 2022-04-05

**Authors:** Xiaojie Yu, Yuanyuan Bai, Jiangfeng Yang, Xiaokun Zhao, Lei Zhang, Jing Wang

**Affiliations:** ^1^College of Animal Science and Technology, Hebei North University, Zhangjiakou, China; ^2^Hebei Mutton Sheep Innovation Strategic Alliance, Zhangjiakou, China

**Keywords:** estrous synchronization, Hu sheep, lambing rate, twinning lamb rate, drug costs

## Abstract

The purpose of this study is to compare five protocols of estrous synchronization for Hu ewes to obtain the most effective and economical protocol, to apply the advantageous scheme in large-scale sheep farming. Healthy multiparous Hu ewes (*n* = 150) were randomly divided into five groups, and all ewes were administered fluorogestone acetate (FGA, 45 mg) vaginal sponge. The sponges of the first three groups (Groups I, II, and III) were removed on the 11th day, and 0.1 mg of PGF_2α_ was injected intramuscularly on the ninth day. Group I received 6 μg of gonadotropin-releasing hormone (GnRH) by intramuscular injection at 36th h after withdrawal of the sponge. Group II was injected 330 IU of pregnant mare serum gonadotropin (PMSG) on the ninth day. The combination of 6 μg of GnRH and 330 IU of PMSG was treated in Group III at the same time as Group I and Group II. The sponges of the latter two groups (Groups IV and V) were removed on the 13th day, and 330 IU of PMSG was injected intramuscularly simultaneously. PGF_2α_ (0.1 mg) was administered on the 12th day in Group IV. All ewes were detected for estrus at 24, 36, 48, 60, and 72 h after the sponge removal. The loss of sponge and vaginitis was recorded when the sponge was withdrawn. Cervical artificial insemination (AI) was performed with fresh semen of Dorper rams diluted with skimmed milk. After 30 days of insemination, the conception was detected with a veterinary B-ultrasound scanner. The lambing status of all ewes and the cost of drugs for estrous synchronization in each group were recorded. The results showed the following: (1) on the whole, the average percentage of estrous ewes in the period of 24–36 h and 36–48 h after removal was significantly higher than other three periods and that of the period of 60–72 h was significantly lower than the first three periods after removal; (2) there was no significant difference in percentages of estrous ewes in any of the five time periods, sponge loss rate, vaginitis rate, total percentage of estrous ewes, conception rate, single lambing rate, twinning rate, and multiple lambing rate of ewes among five protocols; (3) total percentage of estrous ewes and conception rate were more than or equal to 80% in the Groups II and III, and the twinning lamb rate of the Group II protocol was 70%; (4) there was no difference in lambing rate of ewes among Groups II, III, IV, and V; (5) the Group III had the highest drug cost of 22.5 CNY. In conclusion, considering the lambing rate, twinning lamb rate, and drug cost for estrous synchronization, Group II was the most advisable for application and promotion in large-scale sheep farms among these five protocols of estrus synchronization.

## Introduction

In mutton sheep breeding, natural estrus is still an inherent mode. Estrous synchronization is an important reproductive technique to improve the utilization of ewes by shortening the generation interval. Previous studies have shown that estrous synchronization can effectively improve the reproductive performance of sheep, shorten the interval of estrus, and increase the pregnancy rate and lambing rate (1–3). In large-scale breeding farms, estrous synchronization can minimize the effects of seasonal factors on the ewe reproduction, extend the breeding season of ewes, adjust the delivery time of ewes, shorten the lambing cycle, achieve two to three deliveries in a 2-year period (4, 5), thereby reducing the economic cost of breeding, increasing the number of lambs, and obtaining more economic benefits (6, 7).

In the northern part of Hebei, China, Small-tailed Han sheep is the main sheep breed raised on a large scale. Hu sheep, a new breed, was introduced in 2018. Hu sheep can undergo estrous cycles year-round, and it has the advantages of early maturity, two births a year, more lambs per litter, fast growth and development, and improved efficiency of meat production. Hu sheep are raised in this area mainly for breeding to supply lambs to the market. After the use of estrous synchronization, the ewes can undergo natural mating or artificial insemination (AI). To reduce the number of rams and decrease the cost of feeding, AI has been widely used in the estrous synchronization of ewes (8–10). Estrus synchronization and AI are essential assisted reproductive technologies for sheep. Estrus synchronization can make ewes ovulate synchronously and, then through timed AI, make ewes produce lambs synchronously. These technologies allow lambs to enter the market in batches. After the estrus synchronization, the ewes are imposed on AI irrespective of whether the estrus is detected or not. This approach not only saves time but also contributes to improving the conception rate of the ewes (11). AI includes transcervical AI and laparoscopic AI. Laparoscopic AI directly delivers sperm to the fertilization site of the ewe, which reduces the consumption of sperm and greatly improves the conception rate of ewes. Nevertheless, compared with transcervical AI, laparoscopic AI has higher operating costs. Fresh semen, cooled semen, and frozen semen can be selected in AI. The most ideal is of course fresh semen, but frozen semen was the more desirable choice when many influential factors remained such as limitation by time and location. Previous research has demonstrated cryopreservation reduces sperm quality (12). Moreover, the preservation of frozen semen should meet high technical requirements. Regardless, the use of frozen semen breaks the limitations of estrus time of sheep and the geographical location of different breeds, which is beneficial to breed improvement. In this study, fresh semen was used.

At present, there are many hormonal schemes for synchronization of estrus in sheep, such as progestogen vaginal sponge, prostaglandin (PG), and its structural analogs by intramuscular injection (13, 14). The progestogens that have been used include progesterone (P_4_), fluorogestone acetate (FGA), and medroxyprogesterone acetate (MAP). The protocol of progestogens vaginal sponge can be divided into medium long-term treatment (9–16 days) and short-term treatment (5–7 days). Studies have shown that long-term use (11 days) of P_4_ sponge significantly increased the conception rate of ewes compared with short-term use (7 days) (5). The onset of estrus of ewes using Controlled Internal Drug Release (CIDR) for a long time (14 days) was earlier than that of ewes using CIDR for a short time (5, 6, and 7 days) (*P* < 0.05) (15). Another research has shown that the estrus of ewes with the FGA sponge for 14 days was earlier than that of ewes using the FGA sponge for 6 or 7 days (*P* < 0.01) (16). Altineki and Koyuncu (17) studied the effect of FGA vaginal sponge on estrus and lambing of Merino ewes and concluded that the effect of medium-term (10 days) and long-term (14 days) treatments was better than that of short-term treatment (7 days).

PG and their structural analogs are effective luteolysis agents, and they have been widely used in the estrous synchronization of ewes (18, 19). They have advantages including fast metabolism through the lungs, less residue, and low risk of potential environmental pollution (20, 21). They were usually used by intramuscular injection to induce synchronized estrus in the breeding season (22, 23). PG only is used when the ewes are in the estrus cycle because they can dissolve the corpus luteum in the luteal phase of the estrus cycle. It also limits the application of timed AI when two PG injections induce estrus synchronization. In non-breeding seasons, the combined use of progestogen and PG has a good effect on inducing synchronization estrus for ewes (24, 25). PG is usually administered before the vaginal sponge is withdrawn. Martinez-Ros et al. (26) have shown that 63.6% of ewes were in estrus when PGF_2α_ was used before FGA vaginal sponge, and 90.9% of ewes were in estrus when FGA vaginal sponge was used before PGF_2α_. Hence, FGA vaginal sponge was used before PGF_2α_ in this study.

Pregnant mare serum gonadotropin (PMSG) has been widely used for estrus synchronization in ewes. PMSG has the dual activities of follicle-stimulating hormone (FSH) and luteinizing hormone (LH), and it acts directly on the ovary. PMSG can improve the reproductive performance of ewes including inducing superovulation (27) and increasing the pregnancy rate (28). The difference of estrus interval after the removal of the sponge can be reduced by PMSG to achieve the consistency of estrus time of ewes. Gonadotropin-releasing hormone (GnRH) is a hormone secreted by the hypothalamus, whose main physiological role is to stimulate the release of FSH and LH from the anterior pituitary. The research has proved GnRH is the main regulatory factor of reproduction (29). The function of GnRH to induce ovulation has been used in ruminant reproduction (30). In recent years, some researchers have tried to use GnRH instead of PMSG for synchronized estrus in sheep and achieved favorable results (31). PMSG and GnRH were compared in the estrus synchronization of Hu ewes in this study.

On the basis of the previous studies, this study hypothesized that (1) the effects of PMSG and GnRH are similar on the synchronization of estrus in Hu ewes and that (2) the FGA vaginal sponge of long-term use (11 and 13 days) has the same effect on the synchronization of estrus in Hu ewes. The objective of this study is to obtain the most practical protocol by comparing five treatments of estrous synchronization for improving Hu sheep breeding.

## Materials and Methods

### Animals

This study was conducted from April 30, 2019, to November 16, 2019, in a sheep farm in Zhangjiakou, PR China (latitude 40°37′N, longitude 115°8′E, altitude 680 m). Healthy multiparous Hu ewes (*n* = 150) weighing 45.23 ± 5.56 kg were divided into five groups (*n* = 30 in each group) randomly. It is the second or third parturition of ewes in this study. All ewes gave birth normally without dystocia and stillbirth. All lambs were healthy when they were born, and they received colostrum from ewes in time. Every six ewes lived in the same pen. Ewes of each group were conducted in different estrus synchronization protocols. The semen came from 15 healthy Dorper rams that were older than 2 years old. Each ram was 120–130 kg with strong sexual desire. Each ram was fed twice with 1.5 kg of forage, 1.5 kg of concentrate, 0.25 kg of carrots, and two eggs every day at 7:30 am and 6:30 pm. All sheep were house-fed and drank freely. All experimental procedures involving animals in this study were approved by the Welfare Ethics Committee of the Laboratory Animal Center of Hebei North University, Hebei, PR China.

### Protocols of Estrus Synchronization

**Figure 1:**
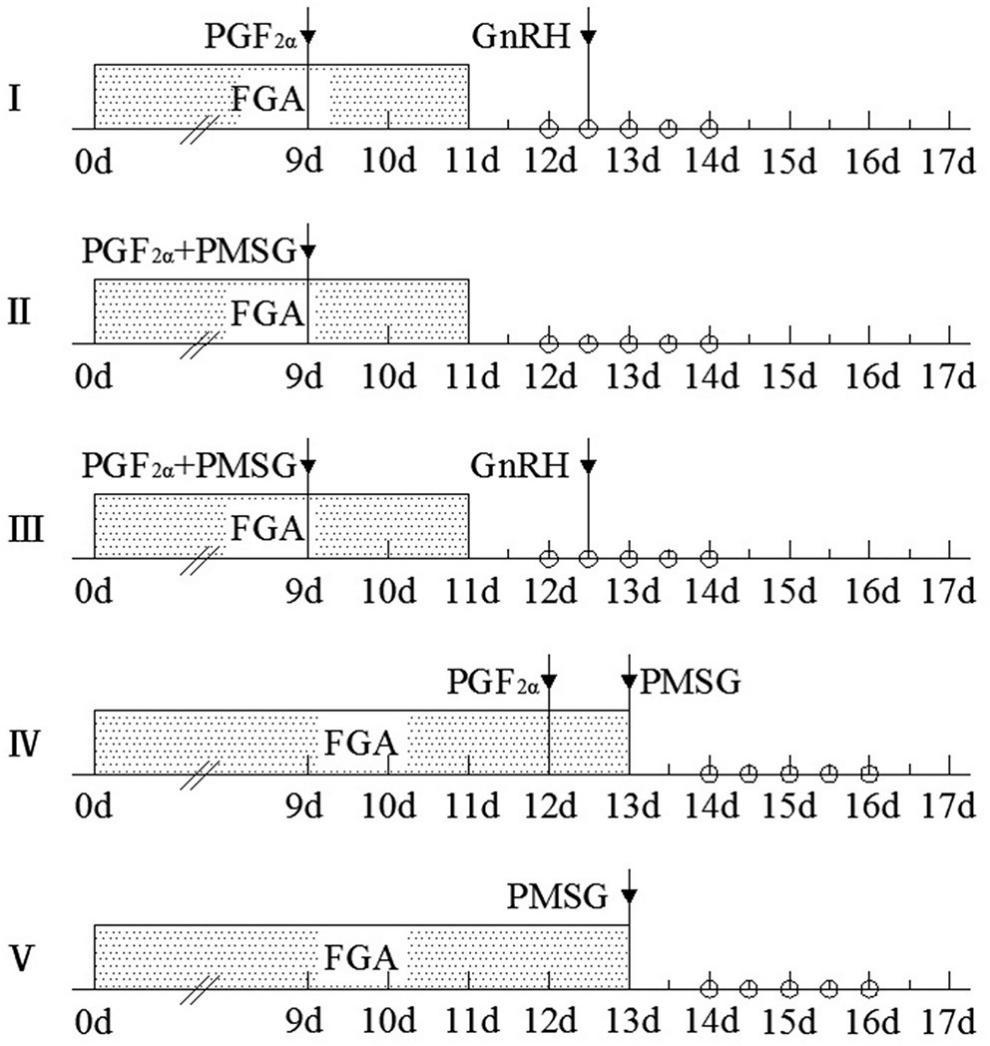
Five protocols of estrous synchronization and the time frame of treatments in ewes. The beginning of fluorogestone acetate (FGA) use was recorded as 0 days, and shadow represents the continuous use of the FGA vaginal suppository. The time indicated by the arrow is the specific time of the use of various hormones in each protocol. FGA vaginal suppository contained 45 mg of fluorogestone acetate, and the intramuscular dose of PGF_2α_, PMSG, and GnRH was 0.1 mg, 330 IU, and 6 μg, respectively. “°” refers to the time points of 24, 36, 48, 60, and 72 h after the sponge removal, and the number of estrous ewes was recorded at these time points.

All ewes were treated with FGA (45 mg) vaginal sponge (SYNCRITE-45 Vaginal Sponge, Animal Health Supplies, 37 Charles St., Ascot Vale, VIC, Australia). Before using the FGA sponge, to prevent vaginitis of ewes, 30 sponges and 160 IU of penicillin sodium were placed in the container at the same time, and the container was shaken for 3 min to make the penicillin sodium spread evenly on the surface of the sponges. The sponges of the first three groups (Groups I, II, III) were removed on the 11th day, and 0.1 mg of PGF_2α_ was injected intramuscularly on the ninth day. Group I received 6 μg of GnRH by intramuscular injection at 36th h after withdrawal of the sponge. Group II was injected 330 IU of PMSG on the ninth day. The combination of 6 μg of GnRH and 330 IU of PMSG was treated in Group III at the same time as Groups I and II. The sponges of the latter two groups (Groups IV and **V**) were removed on the 13th day, and 330 IU of PMSG was injected intramuscularly simultaneously. PGF_2α_ (0.1 mg) was administered on the 12th day in Group IV. PGF_2α_, PMSG, and GnRH were purchased from Ningbo Second Hormone Factory, Cixi City, Zhejiang Province, PR China. The five protocols of estrous synchronization and the time frame of treatments were shown in Figure 1.

### Semen Collection, Evaluation, Dilution, and Artificial Insemination

Semen was collected from 15 Dorper rams (older than 2 years old) using an artificial vagina. The average ejaculation volume of each ram was 0.8–1.2 ml. The appearance of semen is milk-white and rolling up and down like clouds. Sperm motility was observed and the semen with sperm motility over 80% were permitted to use. All permitted semen was mixed. The semen was extended with skimmed milk (semen:skimmed milk = 1:3) to the final sperm concentration was 4 × 10^8^ sperm/ml. The ewes were artificially inseminated at 24, 36, and 48 h after the sponge removal. Cervical AI was operated by the same skilled technician using an insemination instrument (Henan Yuzhu Agriculture and Animal Husbandry Technology Co. LTD, Zhengzhou, China) with a light source. Diluted semen (0.2 ml) was inseminated into the cervical orifice at 0.5–1.0 cm in depth (32). The skimmed milk was purchased from Inner Mongolia Yili Industrial Group Co., Ltd.

### Estrus Identification and Monitoring for Reproductive Performance of Ewes

Estrus of ewes was detected twice a day by aproned rams (aproned rams:ewes to be tested = 1:10). Ewes were determined to be in estrus when they exhibited a standing reflex in response to the aproned ram. The number of estrous ewes was recorded at 24, 36, 48, 60, and 72 h after the removal of the suppository, and the percentage of estrous ewes in different periods to the total number of estrous ewes was calculated.

In addition, the loss of sponge and vaginitis was identified at intravaginal sponge withdrawal. Placement and removal of the sponge, the assessment of the sponge loss, and the identification of vaginitis were always performed by the same researcher. The state of foul smell and purulent discharge from the ewe's vagina was recorded as vaginitis. The loss rate of sponge, vaginitis rate, total percentage of estrous ewes, and conception rate in each group were calculated. After 30 days of insemination, conception was identified by a transabdominal B-mode ultrasound scanner (HS-101V, Honda Electronics Co., Ltd. Tokyo, Japan) equipped with a 3.5-MHz abdominal convex array probe (HCS-136C). Pregnant ewes were characterized by the detection of embryonic vesicles by the scanner.

The number of lambing ewes, the number of live lambs, and the number of ewes with single, twins, and multiple lambs of each group were recorded. Vaginitis rate (%) = number of ewes exhibiting vaginitis/number of ewes treated with intravaginal sponge ×100, conception rate (%) = number of pregnant ewes/number of mating ewes ×100, lambing rate (%) = number of ewes giving birth/number of mating ewes ×100, single lamb rate (%) = number of ewes with single lamb/number of ewes giving birth ×100, twinning lamb rate (%) = number of ewes with twins/number of ewes giving birth ×100, multi-lamb rate (%) = number of ewes birthing triple or more lambs/number of ewes giving birth ×100.

### Calculation of Drug Cost for Synchronized Estrus

The price of all drugs was based on the market price at the time that this study was conducted. A bag of FGA sponges costs 550 CNY and can be used for 50 ewes. The price of the FGA sponge was 11 CNY per ewe. One box of PMSG is 120 CNY containing five vials and 1,000 IU of PMSG in each vial. One vial can be used for three ewes, and one box of PMSG can be used for 15 ewes. The cost of PMSG was 8 CNY per ewe. The cost of one box of PGF_2α_ (0.2 mg × 10 vials) was 50 CNY. One ewe was injected with 0.1 mg of PGF_2α_. One box of PGF_2α_ can be used for 20 ewes, with an average of 2.5 CNY per ewe. The cost of one box of GnRH (25 μg × 10 vials) was 30 CNY. One ewe was treated with 6 μg of GnRH. One box of GnRH was used for 40 ewes, with an average of 0.75 CNY per ewe.

### Statistical Analysis

All data were analyzed using SPSS statistical software (version 21.0, SPSS Inc., Chicago, IL, USA). Chi-square analysis was used to compare the loss rate of sponge, vaginitis rate, percentage of estrous ewes, conception rate, single lamb rate, twinning lamb rate, and multi-lamb rate of ewes among five protocols. The lambing rate of ewes among five treatments and the percentage of estrous ewes in different time periods were compared by one-way analysis of variance, and Tamhane's T2 of multiple comparisons was used when equal variances were not assumed among groups. Data were considered significant at *P* < 0.05.

## Results

### Percentage of Estrous Ewes in Different Time Periods

To avoid missing ewes in heat, all ewes in heat within 72 h after the withdrawal of the sponge were recorded. As shown in Table 1, the 72 h after the removal of the sponge were divided into five time periods. In the same time period, there was no significant difference (*P* > 0.05) in the percentage of estrous ewes among the five treatments. The average percentage of estrous ewes was significantly different (*P* < 0.05) in different time periods. The percentage of estrous ewes in 24–36 h and 36–48 h was significantly higher than that of the other three time periods (*P* < 0.05), and the difference in the percentage of estrous ewes between these two time periods was not significant (*P* > 0.05). The percentage of estrous ewes within 60–72 h was significantly lower than that of the first three time periods (*P* < 0.05).

**Table 1:** Percentage of estrous ewes in different time periods (%).

**Time periods**	**I**	**II**	**III**	**IV**	**V**	**χ^2^**	***P***	**Mean**
0–24 h	9.52 (2/21)	8.00 (2/25)	11.54 (3/26)	8.33 (2/24)	13.04 (3/23)	0.140	0.708	10.09 ± 2.16^b^
24–36 h	42.86 (9/21)	36.00 (9/25)	42.31 (11/26)	33.33 (8/24)	34.78 (8/23)	0.320	0.571	37.86 ± 4.42^a^
36–48 h	33.33 (7/21)	44.00 (11/25)	34.62 (9/26)	41.67 (10/24)	39.13 (9/23)	0.063	0.802	38.55 ± 4.54^a^
48–60 h	9.52 (2/21)	8.00 (2/25)	3.85 (1/26)	12.50 (3/24)	8.70 (2/23)	0.032	0.858	8.51 ± 3.12^bc^
60–72 h	4.76 (1/21)	4.00 (1/25)	7.69 (2/26)	4.17 (1/24)	4.35 (1/23)	0.002	0.963	4.99 ± 1.53^c^

*Different lowercase letters on the shoulder of the data in the same column indicate that there is a significant difference (P < 0.05) in the average percentage of estrous ewes of five protocols in different time periods*.

### Reproductive Performance of Hu Ewes With Five Protocols of Estrous Synchronization

The reproductive performance of ewes is shown in Table 2. There were no significant differences (*P* > 0.05) in the loss rate of sponge, vaginitis rate, total percentage of estrous ewes, conception rate, single lamb rate, twinning lamb rate, and multi-lamb rate of ewes among the five protocols. The total percentage of estrous ewes and conception rate is either ≥80% in Groups II and III. The lambing rate of ewes of Group III was significantly higher than that of Group I (*P* < 0.05), and there was no significant difference in the lambing rate of ewes of the latter four groups.

**Table 2:** Reproductive performance of Hu ewes with five protocols of estrous synchronization (%).

**Indexes**	**I**	**II**	**III**	**IV**	**V**	**χ^2^**	**P**
Loss rate of sponge	0.00 (0/30)	3.33 (1/30)	0.00 (0/30)	3.33 (1/30)	6.67 (2/30)	2.041	0.153
Vaginitis rate	0.00 (0/30)	0.00 (0/30)	3.33 (1/30)	6.67 (2/30)	0.00 (0/30)	0.676	0.411
TP of estrus^#^	70.00 (21/30)	83.33 (25/30)	86.67 (26/30)	80.00 (24/30)	76.67 (23/30)	0.182	0.670
Conception rate	71.43 (15/21)	80.00 (20/25)	80.77 (21/26)	75.00 (18/24)	73.91 (17/23)	0.002	0.963
Lambing rate	147.62^b^ (31/21)	192.00^ab^ (48/25)	207.69^a^ (54/26)	175.00^ab^ (42/24)	165.22^ab^ (38/23)	—	<0.05
Single lamb rate	20.00 (3/15)	5.00 (1/20)	4.76 (1/21)	16.67 (3/18)	17.65 (3/17)	0.173	0.677
Twinning rate	53.33 (8/15)	70.00 (14/20)	66.67 (14/21)	55.56 (10/18)	64.71 (11/17)	0.014	0.905
Multi-lamb rate	26.67 (4/15)	25.00 (5/20)	28.57 (6/21)	27.78 (5/18)	17.65 (3/17)	0.198	0.656

# *“TP of estrus” represents “total percentage of estrous ewes”*.

*The different lowercase letters on the shoulder of the data in the same row indicate that there is a significant difference in the lambing rate of ewes (P < 0.05) of five protocols of estrus synchronization*.

### Drug Cost of Five Protocols of Estrus Synchronization

Table 3 shows the drug cost of estrous synchronization of a single ewe of five protocols. The drug cost of Group III was the highest, which is CNY 22.5. The drug cost of Group I was the lowest, which is CNY 14.5. The cost of Groups II and IVwas the same.

**Table 3:** Drug cost of estrus synchronization of single ewe of five protocols (CNY).

**Protocols**	**FGA**	**PMSG**	**PGF_**2α**_**	**GnRH**	**Total**
I	11.00	—	2.50	0.75	14.25
II	11.00	8.00	2.50	—	21.50
III	11.00	8.00	2.50	0.75	22.25
IV	11.00	8.00	2.50	—	21.50
V	11.00	8.00	—	—	19.00

## Discussion

### Estrus Performance of Ewes

Biehl et al. (5) have shown that the estrous behavior of ewes mainly appeared within 48 h after the withdrawal of the progestogen sponge, which is the same as the results of this study. Other studies have shown that there was no significant difference in the total estrus response of ewes with different estrous synchronization treatments (33, 34), which is the same as the results of this study. PMSG acts directly on the ovary, effectively promoting the growth and development of follicles and facilitating the estrous response of ewes. PMSG metabolizes in animals slowly. The excessive use of PMSG could lead to the coexistence of corpus luteum and follicles in the ovary of ewes. It will affect implantation and conception after insemination or embryo transfer and even cause ovarian cysts in ewes. GnRH and its synthetic analogs can stimulate the anterior pituitary to release FSH and LH, which can effectively induce ovulation and shorten the interval from estrus to ovulation. The results of this study showed that PMSG and GnRH had no difference in promoting synchronized estrus of Hu ewes. The results proved that the first hypothesis of this study was established.

The percentage of estrous ewes treated with progestogen sponge for a long term (14 days) was significantly higher than that of ewes treated for 7 and 10 days (*P* < 0.05) (17). In this study, there was no significant difference in the total percentage of estrous ewes treated with different protocols of estrus synchronization, and there was no significant difference in the proportion of estrous ewes in any one of the five time periods within 72 h after withdrawal of the sponge among five protocols. It may be because the 11 and 13 days are both long-term treatments, and the time difference of 2 days is not enough long to cause the difference in the percentage of estrous ewes. The results proved that the second hypothesis of this study was established.

From the perspective of shortening the breeding cycle and saving breeding costs, it is recommended to use the FGA sponge for 11 days, namely, the protocols of Groups I, II, and III. Although there was no significant difference in the total percentage of estrous ewes in different schemes, the total percentage of estrous ewes in the scheme of Group I was only 70%, whereas it was both more than 80% in the schemes of Groups II and III. Therefore, two schemes of Groups II and III could be recommended.

### Conception and Lambing of Ewes

Estrus, conception, and lambing are important indexes to measure the reproductive efficiency of sheep. Quintero-Elisea et al. (35) have shown that there was no significant difference in the conception rate of ewes in different protocols of estrous synchronization with FGA-based combined with PMSG (*P* > 0.05), and there was no significant effect on the conception rate of ewes with the use of PMSG at different time points, which were consistent with the results of this study. Although there was no significant difference in the conception rate of ewes among different schemes of estrous synchronization in this study, only two schemes of Groups II and III have a conception rate equal to or >80%.

It was reported that the lambing rate of ewes treated with FGA (30 mg) for 14 days was significantly higher than that of ewes treated for 7 days (*P* < 0.05) (17), indicating that the long period treatment of the FGA intravaginal sponge was conducive to lambing rate of ewes. It is worth noting that the breeders usually wish the twinning lamb because compared with multi-lamb, twinning lamb has larger birth weight, easier survival, and better growth (36), which is especially conducive to the breeding and management of large-scale sheep farms. Although there was no significant difference in the twinning lamb rate of the five protocols of estrous synchronization, only Group II had the twinning lamb rate of 70%, and that of the other schemes was all <70%. The results showed it was appropriate that the administered dosage and time of all drugs in GroupII. Therefore, the scheme of Group II was recommended in large-scale sheep farms.

### Loss Rate of Sponge and Vaginitis Rate of Ewes

Regarding the loss rate of sponge of ewes, the results of previous studies are not consistent. Fonseca et al. (37) and Husein et al. (38) have shown that the longer the treatment time of the vaginal sponge in ewes, the more likely the sponge was lost, indicating that the treatment time is one of the influencing factors for the loss of the sponge. However, Swelum et al. (39) concluded that there was no significant difference in the loss rate of sponge in ewes with a different treatment time of the intravaginal estrus device (3, 6, 9, and 12 days). The results of this study showed that there was no significant difference in the loss rate of sponge of ewes among the five treatments of estrous synchronization, which was consistent with the latter. However, it should be noted that the loss rate of the sponge showed an upward trend when the sponge was used for 13 days.

Studies have shown that local vaginitis and alterations of vaginal flora were present after the ewes were treated with the intravaginal sponge (40, 41). Gram-negative bacteria and gram-positive bacteria appeared in the vagina after using the vaginal progestogen estrous device (42). Manes et al. revealed that, after the use of intravaginal sponges, the vaginal wall of treated ewes showed epithelial hyperplasia and hypertrophy, and perivascular inflammatory cell infiltration (43). These histological and cytological changes are similar to those which occurred in other inflammatory processes of the reproductive tract and could have a negative impact on the fertility of ewes for estrus synchronization. Martinez-Ros et al. have indicated that abnormal vaginal discharge occurred after the ewes were administered with intravaginal sponges for estrus synchronization. More ewes had purulent and hemorrhagic discharges, which were administered by the intravaginal sponge for the long term (44). Purulent and hemorrhagic discharges were typical features of vaginitis. Vaginitis has little effect on the fetus in the early stages of pregnancy but may cause fetal infection due to the presence of vaginal bacteria during the birth of lambs. Therefore, to reduce the inflammatory reaction of the vagina of ewes, antibiotics are usually used when using vaginal progestogens estrous device to induce estrous synchronization (45). The amount of mucus (*P* < 0.01) and odor (*P* < 0.001) were reduced in the antibiotic group vs. the control group when the ewes were treated by the vaginal device for estrous synchronization (46). In this study, vaginal suppositories were used for 11 and 13 days, which are long-term programs, so antibiotics were used to prevent the occurrence of vaginitis in ewes. Antibiotics can prevent the occurrence of vaginitis to a certain extent, but they cannot completely inhibit the occurrence of vaginitis. The results of this study are consistent with previous research. Although antibiotics were used, some ewes still showed vaginitis, and there was no significant difference in the vaginal inflammation rate of ewes among five protocols of estrous synchronization.

### Drug Cost of Estrus Synchronization

In practice, the drug cost is one of the main considerations of farmers, and farmers always want to invest less and produce more. In this study, the drug cost of the protocol of Group I was the lowest, but the total percentage of estrous ewes and conception rate is not dominant. The schemes with the total percentage of estrous ewes and conception rate equal to or >80% were Groups II and III. However, the drug cost of Group III was the highest. Therefore, the protocol of Group III is not recommended in large-scale farms. Although the drug costs of Groups II and IV are the same, the evaluation of the conception rate, lambing rate, twinning rate, and vaginitis rate of Group II is more competitive. Hasani et al. (2) reported that the most expensive protocol of estrus synchronization did not have the significant performance of estrus and reproduction, and the estrous performance of ewes with the moderate price protocol was more consistent than that of expensive synchronization protocol, and the conception rate and lambing rate of ewes were higher in estrus synchronization scheme with moderate cost, which was consistent with the results of this study.

## Conclusions

Considering the percentage of estrous ewes, lambing rate, twinning lamb rate, and drug cost, the protocol of Group II can be recommended to put into use in large-scale Hu sheep farms.

## Data Availability Statement

The original contributions presented in the study are included in the article/supplementary material, further inquiries can be directed to the corresponding author.

## Ethics Statement

The animal study was reviewed and approved by Welfare Ethics Committee of the Laboratory Animal Center of Hebei North University, Hebei, PR China.

## Author Contributions

JW: funding acquisition, project administration, supervision, conceptualization, methodology, investigation, writing—review, and editing. XY: investigation, data analysis, writing—review, and editing. YB: investigation, data analysis, and writing—original draft preparation. JY and XZ: investigation and software. LZ: investigation. All authors have read and agreed to the published version of the manuscript.

## Funding

This research was funded by Key Research and Development Plan Program of Hebei Province, China (Grant Nos: 18236609D and 20326629D), Innovative Capacity Improvement Program of Hebei Province, China (Grant No: 20536601D), and the Excellent Going Abroad Experts' Training Program in Hebei Province, China.

## Conflict of Interest

The authors declare that the research was conducted in the absence of any commercial or financial relationships that could be construed as a potential conflict of interest.

## Publisher's Note

All claims expressed in this article are solely those of the authors and do not necessarily represent those of their affiliated organizations, or those of the publisher, the editors and the reviewers. Any product that may be evaluated in this article, or claim that may be made by its manufacturer, is not guaranteed or endorsed by the publisher.
